# Real-world treatment patterns and outcomes in patients initiating lurasidone for the treatment of schizophrenia in Europe

**DOI:** 10.1192/j.eurpsy.2022.537

**Published:** 2022-09-01

**Authors:** A. Jones, M. Sargeant, M. Andiappan

**Affiliations:** 1 CNX Therapeutics, Psychiatry, London, United Kingdom; 2 Hywel Dda University Health Board, St David’s Hospital, Wales, United Kingdom; 3 OPEN Health, Evidence And Access, Marlow, United Kingdom

**Keywords:** schizophrénia, Antipsychotics, lurasidone, observational

## Abstract

**Introduction:**

Lurasidone is a second-generation antipsychotic shown to have a lower risk of weight gain and a lower incidence of metabolic adverse events compared with some medications in the same class.

**Objectives:**

To describe treatment patterns, clinical outcomes and adverse drug reactions (ADRs) over 12 months following lurasidone initiation in patients with schizophrenia.

**Methods:**

This was a multi-centre observational study involving data collection from patients’ medical records, conducted in seven mental health centres in the United Kingdom (UK) and Switzerland. The study included patients aged ≥18 years who initiated lurasidone after 1 January 2016 for the treatment of schizophrenia. Data were collected from medical records both retrospectively and prospectively using a standardised data collection form. Data collected included patient characteristics, treatment history, lurasidone regimens, clinical outcomes and ADRs.

**Results:**

Forty-eight patients participated in the study. The median (interquartile range [IQR]) age at lurasidone initiation was 33.5 (25.5-50.3) years and 31 (65%) patients were male. The median (range) lurasidone starting dose was 37 mg daily (9.3–148 mg). Thirty-eight (79%) patients continued lurasidone for the entire 12-month follow-up period. Among the 14 (29%) patients with documented relapse, the median (IQR) time to relapse was 3.4 (1.5–7.9) months. Five ADRs were recorded in patient notes judged as related to lurasidone: agitation, nausea, akathisia, somnolence and vomiting (one patient each).

**Conclusions:**

In this real-world study of patients with schizophrenia in the UK and Switzerland, 79% of patients continued lurasidone for at least 12 months, and ADRs were reported rarely in patient notes.

**Disclosure:**

This study was sponsored by CNX Therapeutics Ltd (formerly Sunovion Pharmaceuticals Europe Ltd). AJ is an employee of CNX Therapeutics. MA is an employee of OPEN HEALTH who was contracted by CNX Therapeutics for data analysis and medical writing.

